# Optic neuropathy as an early manifestation of granulomatosis with polyangiitis: a case report and literature review

**DOI:** 10.3389/fmed.2025.1515622

**Published:** 2025-02-18

**Authors:** Yukang Kim, Tonghoon Woo, Sun-Uk Lee, Euyhyun Park, Hyun-Jin Shin, Jongmin Sim, Gerard Jounghyun Kim, Ji-Soo Kim

**Affiliations:** ^1^Neurotology and Neuro-Ophthalmology Laboratory, Korea University Medical Center, Seoul, Republic of Korea; ^2^Department of Neurology, Korea University Medical Center, Seoul, Republic of Korea; ^3^Department of Otorhinolaryngology-Head and Neck Surgery, Korea University Medical Center, Seoul, Republic of Korea; ^4^Department of Ophthalmology, Konkuk University Medical Center, Seoul, Republic of Korea; ^5^Department of Pathology, Korea University Anam Hospital, Korea University College of Medicine, Seoul, Republic of Korea; ^6^Department of Computer Science and Engineering, Korea University, Seoul, Republic of Korea; ^7^Department of Neurology, Seoul National University College of Medicine, Seoul, Republic of Korea; ^8^Dizziness Center, Clinical Neuroscience Center, Department of Neurology, Seoul National University Bundang Hospital, Seongnam, Republic of Korea

**Keywords:** optic neuritis, granulomatosis with polyangiitis, Wegener’s granulomatosis, vasculitis, ischemic optic neuropathy

## Abstract

**Introduction:**

Ophthalmic involvement occurs in up to 40% of patients with granulomatosis with polyangiitis (GPA), usually confined to the anterior segment. Herein, we describe patients presenting with optic neuropathy as an early manifestation of GPA, without other signs of ocular or adnexa involvement.

**Methods:**

We report a case of isolated optic neuropathy without other ocular or adnexal involvement and examine the reported clinical features of 17 additional patients through a literature review. We analyzed clinical characteristics and neuro-ophthalmological findings and discuss the clinical implications for the early detection of GPA-associated optic neuropathy.

**Results:**

Among the 17 patients, 10 had optic neuropathy confined to one eye, three exhibited simultaneous bilateral optic neuropathies at initial presentation, and four had unilateral involvement initially; however, the fellow eye was subsequently affected during follow-up. Nine patients had optic neuropathy as the first clinical presentation and no prior diagnosis of GPA (9/17, 53%). Among the 21 eyes (15 patients, excluding two without descriptions), disc edema was observed in five eyes (24%). Visual impairment was often profound; the measurements of 23 affected eyes at the initial presentation showed that the patient’s acuity was to count fingers or worse (14/23, 61%). The final visual outcome was often poor, with significant visual recovery in only eight eyes (8/23, 35%). Other constitutional symptoms or systemic involvements were found in most patients (15/16, 94%), mostly affecting the lung (*n* = 10), sinus (*n* = 9), and pachymeninges (*n* = 8). Furthermore, 88% of the patients (15/17) showed positive results on antineutrophil cytoplastic antibody. Elevated CRP (*n* = 6) or ESR (*n* = 5) was found in 56% of cases.

**Discussion:**

Our case and literature review indicates that optic neuropathy can present in the context of systemic inflammation of GPA, without any other signs of ocular or orbital involvement. Catching other clinical, imaging, and laboratory signs of systemic inflammation is important in cases of GPA-associated optic neuropathy with atypical presentations.

## 1 Introduction

Granulomatosis with polyangiitis (GPA), previously referred to as Wegener’s granulomatosis, is an autoimmune vasculitis affecting small- and medium-sized blood vessels (1). This condition may involve various organs, including the sinuses, nose, throat, lungs, and kidneys, typically presenting as rhinitis, chronic otitis media, pneumonia, or glomerulonephritis. Ophthalmic involvement is observed in up to 40% of patients (2), primarily affecting the anterior segment (2–5). Although rare, the optic nerve can also be affected, mostly due to granulomatous lesions or direct spread of inflammation from the sinuses (2).

We recently encountered an unusual case of acute unilateral optic neuropathy as the first presentation of GPA. The diagnosis posed a challenge, as imaging revealed no granulomatous inflammation, and there was no evidence of ocular or ocular adnexal involvement. This implies that optic neuropathy can result from *in situ* pathology in the optic nerve, aside from direct compression from inflammation in GPA, as is conventionally known. Defining these patients is important because treatment considerations are different for GPA optic neuropathy versus demyelinating optic neuritis. Thus, we report the details of this case and provide a systematic review of the literature on optic neuropathy without any other signs of ocular or ocular adnexal involvement as a rare presentation of GPA.

## 2 Methods

Written informed consent was obtained from the patient for the publication of any potentially identifiable images or data included in this article.

### 2.1 Case report

#### 2.1.1 History and examination

An 80-year-old female with hypertension and diabetes mellitus presented with an acute decrease in visual acuity in the right eye for 2 days. The patient experienced an unexplained fever and a weight loss of 6 kg in the month prior to presentation. The patient did not report diplopia, ocular pain, headache, or dizziness.

The patient underwent a comprehensive ophthalmic examination with the following pertinent findings. The eyes were orthophoric with intact versions and ductions. Her visual acuity measurements showed light perception in the right eye and 20/40 in the left. Both pupils were round, isocoric, and reactive to light and accommodative stimuli. A relative afferent pupillary defect was observed in the right eye. Other neurologic examinations, including palpation of the preauricular and forehead arteries, were normal. Fundus examination revealed a pallid disc edema in the right eye.

**Figure 1:**
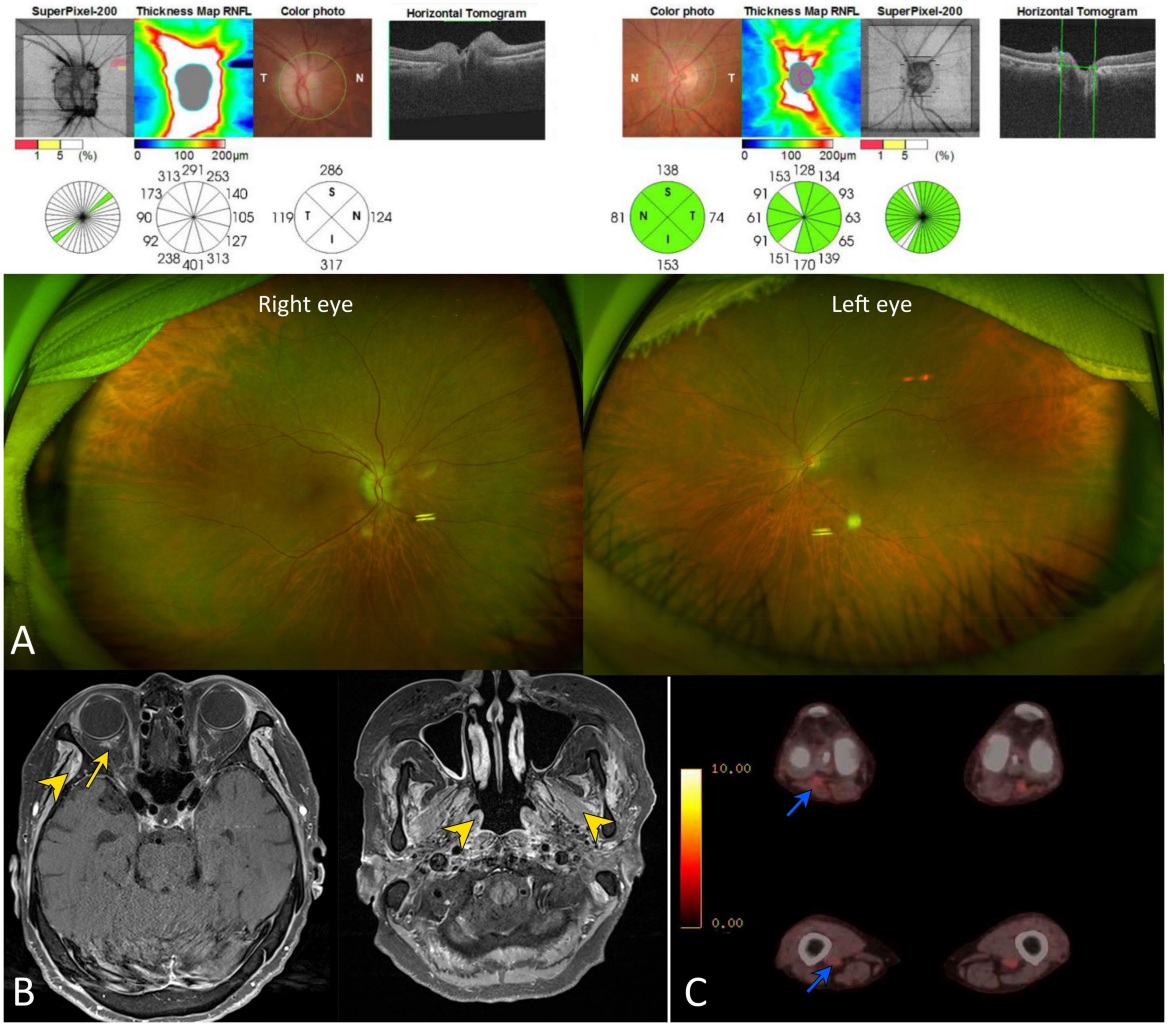
Neuro-ophthalmologic and imaging findings of our patient. **(A)** Scanning laser ophthalmoscopy and optical coherence tomography show diffuse disc swelling in the right eye. **(B)** A gadolinium enhancement was documented around the right optic nerve sheath on gadolinium-enhanced T1-weighted images (yellow arrow). This finding suggests that the posterior ciliary arteries may be affected, secondary to adjacent inflammation. Multifocal enhancements were also found in bilateral temporalis, masseter, and pterygoid muscles (yellow arrowheads). **(C)** Whole-body positron emission tomography (PET) showed an abnormal glucose uptake in the popliteal and femoral arteries (blue arrowheads).

Standard automated perimetry using the Humphrey Visual Field Analyzer (program 24-2 full threshold, white stimulus; Carl Zeiss Meditec, Dublin, United States) showed global depression in the right eye and normal findings in the left eye. The optical coherence tomography (OCT, Cirrus; Carl Zeiss Meditec, Dublin, United States) showed marked thickening of retinal nerve fiber layer in right eye consistent with disc edema (Figure 1A). Fluorescein angiography was not performed because the marginal glomerular filtration rate was 26.6 mL/min/1.73 m^2^. No responses were obtained during right-eye stimulation of visually evoked potentials. The visually evoked potential showed normal response in the left eye.

#### 2.1.2 Ancillary testing

**Figure 2:**
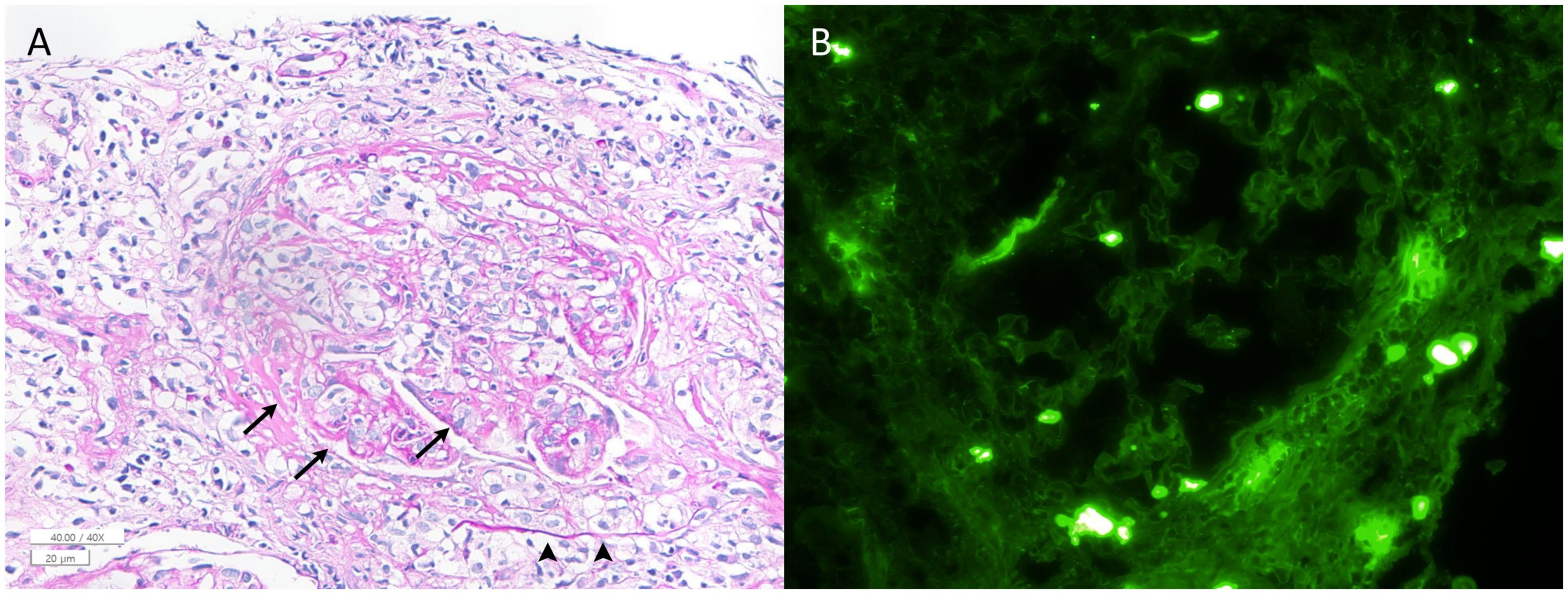
Histologic findings from the kidney biopsy sample. **(A)** A periodic acid–Schiff-stained section shows a glomerulus with a cellular crescent (arrowheads) and fibrinoid necrosis (arrows) (×400). No evidence of granulomatous inflammation was observed in the specimen. **(B)** Immunofluorescence microscopy demonstrates the absence of IgG staining in the glomerulus (×400). Staining for IgA, IgM, C1q, C3, kappa, lambda, and fibrinogen is also negative (image not shown). These pathological findings were consistent with pauci-immune crescentic glomerulonephritis.

Although chest X-ray was unrevealing, chest CT revealed multiple ill-defined nodules and ground-glass opacity less than 1 cm in the right middle lobe. Urine analysis showed proteinuria of 1+. A gadolinium enhancement was found around the right optic nerve sheath on MRI (Figure 1B). Multifocal enhancements were documented in the bilateral temporalis, masseter, and pterygoid muscles, aside from maxillary sinusitis and mastoiditis. Whole-body positron emission tomography showed increased glucose uptake in the popliteal and femoral arteries (Figure 1C). The serum was positive for cytoplasmic antineurtrophil cytoplasmic antibody (cANCA) and negative for perinuclear ANCA (pANCA). The serum titer of anti-myeloperoxidase antineutrophil cytoplasmic antibody increased to 105.0 IU/mL (MPO-ANCA, normal range = 0–2 IU/mL), whereas that of anti-proteinase 3 antineutrophil cytoplasmic antibodies (PR3-ANCA) was normal using an enzyme-linked immunosorbent assay. The erythrocyte sedimentation rate (ESR, 92 mm/h) and C-reactive protein level (CRP, 145.0 mg/L) were elevated. The serum was negative for anti-aquaporin-4, anti-myelin oligodendrocyte glycoprotein, and paraneoplastic antibodies. CSF analysis revealed no leukocytosis or albuminocytologic dissociation but showed positive results for the oligoclonal band. A kidney biopsy documented pauci-immune crescentic glomerulonephritis compatible with the diagnosis of GPA (Figure 2).

#### 2.1.3 Diagnosis

The patient was diagnosed with optic neuropathy associated with GPA. The patient scored 9 points on 2022 American College of Rheumatology/European Alliance of Associations for Rheumatology (ACR/EULAR) classification criteria (5 points for cANCA, 2 points for pulmonary nodule, 1 point for maxillary sinusitis and mastoiditis on MRI, and 1 point for pauci-immune glomerulonephritis on biopsy) (6).

#### 2.1.4 Treatment and clinical course

The patient received intravenous methylprednisolone (1 g/day) for five consecutive days. Intravenous rituximab (1,000 mg) was administered for immunomodulation. Three months later, optic disc pallor was observed in the right eye. Visual acuity measurements showed light perception in the right eye and at 20/25 in the left. Recurrence was not observed during the 2-year follow-up, and immunomodulation was maintained with intravenous rituximab.

### 2.2 Literature search

We performed a literature search using PubMed (up to August 2024). The search keywords included *optic neuropathy*, *optic neuritis*, *Wegener*’*s granulomatosis*, and *granulomatosis with polyangiitis*. We included all patients described in the systematic reviews, clinical studies, and case reports published in English. The references cited in the retrieved articles were also reviewed. The diagnosis of optic neuropathy/neuritis as an isolated manifestation was based on (1) documented optic nerve involvement, (2) documentation and quantification of nerve damage using neuro-ophthalmologic examination and evaluation, and (3) the absence of any other ocular or ocular adnexa abnormalities.

We analyzed the clinical characteristics of the patients: the bilaterality of optic nerve involvement, best-corrected visual acuities recorded at initial presentation and the final visit, and results of neuro-ophthalmologic evaluation and serologic, cerebrospinal fluid (CSF), and magnetic resonance imaging (MRI) findings.

## 3 Results

**Figure 3:**
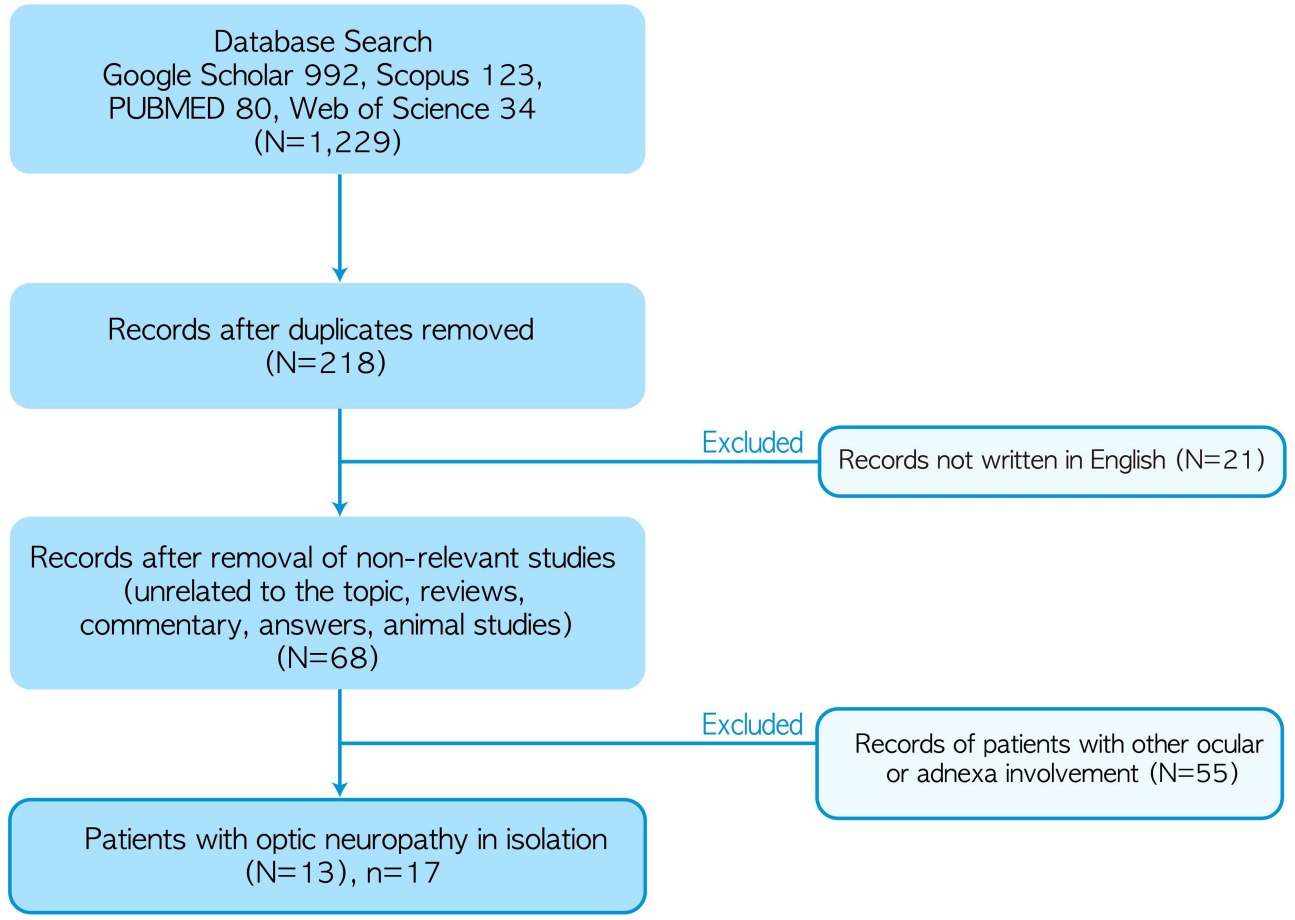
Algorithm for literature review. ^*^Not including our patient.

A comprehensive literature review identified 13 studies describing 17 patients who met the search criteria (6 female, age range: 32–87 years, median age: 64 years, mean age ± standard deviation = 64 ± 14; Figure 3 and Table 1). Fourteen patients were found to have isolated optic neuropathy associated with GPA. Three patients initially presented with optic neuropathy in isolation but subsequently developed strabismus during follow-up due to abducens (*n* = 2), oculomotor (*n* = 2), or trochlear nerve palsy (*n* = 1; Table 1).

**Table 1:** Literature review of the cases presenting with optic neuropathy without other ocular or ocular adnexal involvement in granulomatosis with polyangiitis.

Author, year	Sex/age	Timing of optic nerve involvement	Optic nerve involvement	Fundus findings^a^	Visual acuity^a^		Other organ involvement or vegetative symptom	Serology			
					Initial	Final		MPO-ANCA (IU/mL) or pANCA	PR3-ANCA (IU/mL) or cANCA	CRP (mg/L)	ESR (mm/h)
Our study	F/80	Initial	OD	Pallid disc edema	LP	LP	Kidney, large vessel, fever, weight loss	MPO-ANCA (105.0)	cANCA (1:80)	145	92
Belden et al., 1993 (27)	F/55	During the disease process	OU, sequential (OS → OD)	Normal	FC, HM	FC, FC	Lung, sinus, pachymeninges, skin/connective tissue (nasal septum), colon	Unknown	cANCA (52)	Unknown	72
Duran et al., 2004 (9)	M/67	Initial	OD	Disc edema	Unknown	Normal	Kidney, joint	MPO-ANCA (907)	—	Unknown	75
Monteiro et al., 2005 (8)	M/32	Initial	OU, sequential (OD → OS)	Normal, disc edema	FC, NLP	20/20, 20/30	Lung, sinus, skin/connective tissue (nasal septum), hearing loss, facial nerve	—	cANCA (+)	Unknown	Unknown
Blaise et al., 2007 (28)	F/69	Initial	OU, sequential (OD → OS)	Normal	NLP, LP	LP, LP	Weight loss	pANCA (+)	cANCA (+)	Normal	Normal
Blaise et al., 2007 (28)	F/58	During the disease process	OS	Unknown	LP	LP	Lung, weight loss	—	—	Unknown	Unknown
Purvin and Kawasaki, 2009 (16)	M/53	Initial	OS	Disc hyperemia	20/20	20/20	Lung	pANCA normal	cANCA (12)	Normal	Normal
Huchzermeyer et al., 2013 (29)^b^	F/56	During the disease process	OD	Normal	20/100	20/25	Skin/connective tissue (nasal septum, saddle nose)	Unknown	PR3-ANCA +, cANCA (60)	Unknown	Unknown
Nagaoka et al., 2012 (30)	M/71	Initial	OU	Unknown	NLP, 20/40	Unknown	Lung, pachymeninges	Unknown	PR3-ANCA	Unknown	Unknown
Takazawa et al., 2014 (31)	M/74	Initial	OU, sequential (OD → OS)	Normal	NLP, 20/100	NLP, 20/20	Lung, pachymeninges, kidney, skin/connective tissue (nasal cavity, throat)	Unknown	PR3-ANCA	8.9	Unknown
Takazawa et al., 2014 (31)	M/72	Initial	OU	Normal	NLP, 20/40	Complete resolution	Lung, sinus, pachymeninges, chronic otitis media	Unknown	PR3-ANCA	8.9	>100
Nakajima et al., 2016 (32)	F/87	Initial	OS	Normal	LP	Improvement	Pachymeninges	MPO-ANCA (5.1)	PR3-ANCA (7.1)	9.25	Unknown
Clément et al., 2019 (20)^b^	M/39	During the disease process	Unilateral (eye not specified)	Optic atrophy	20/200	NLP	Lung, sinus, fever, weight loss, joint	—	PR3-ANCA	188	Unknown
Clément et al., 2019 (20)^b^	F/69	During the disease process	Unilateral (eye not specified)	Normal	FC	NLP	Sinus, pachymeninges, joint, pericardium	MPO-ANCA	—	Normal	Unknown
Clément et al., 2019 (20)	M/64	During the disease process	Unilateral (eye not specified)	Normal	20/200	20/32	Sinus, pachymeninges, fever, joint, peripheral nerve	MPO-ANCA	—	Normal	Unknown
Suga et al., 2019 (33)	M/61	During the disease process	OU	Disc edema	20/20, 20/1,000	20/20, 20/2,000	Lung, sinus, fever, weight loss	—	—	8.24	Unknown
Mitsuhashi et al., 2020 (34)	M/60	During the disease process	OS	Disc edema	20/2,000	20/16	Sinus, fever, vestibulocochlear nerve	MPO-ANCA (6.2)	—	Normal	Unknown
Sato-Akushichi et al., 2021 (35)	M/79	Initial	OS	Normal	LP	20/25	Lung, sinus, pachymeninges, kidney	MPO-ANCA (19.5)	—	Normal	31

ANCA, anti-neutrophil cytoplasmic antibody; cANCA, cytoplasmic ANCA; CRP, C-reactive protein; F, female; ESR, erythrocyte sedimentation rate; FC, finger count; HM, hand motion; L, light perception; M, male; NLP, no light perception; OD, right eye; OS, left eye; OU, both eyes; pANCA, perinuclear ANCA.

a OD, OS in case of bilateral involvement.

b These patients initially presented with optic neuropathy in isolation and later developed strabismus during the disease course.

Nine patients presented with optic neuropathy as the first clinical presentation, with no prior diagnosis of GPA (9/17, 53%). Systemic involvement was observed in most cases (15/16, 94%), affecting the lungs (*n* = 10), sinuses (*n* = 9), pachymeninges (*n* = 8), joints (*n* = 4), kidneys (*n* = 3), skin/connective tissue (*n* = 4), inner/middle ear (*n* = 2), pericardium (*n* = 1), colon (*n* = 1), and cranial (*n* = 2) or peripheral nerves (*n* = 1). Six patients (35%) had preceding constitutional symptoms, such as fever (*n* = 4) or weight loss (*n* = 4).

Among the 17 patients, 10 had optic neuropathy confined to either eye, three exhibited simultaneous bilateral optic neuropathy at the initial presentation, and four had unilateral involvement initially; however, the fellow eye was subsequently affected during follow-up, with a time interval of 2 months to 1 year. Among the 21 eyes (15 patients, excluding two lacking description), disc edema was observed in five eyes (24%). The initial visual acuity was usually poor; the measurements of 14 affected eyes showed the patient was only to count fingers (14/23, 61%). Visual field tests revealed various types of visual field defects, including central/cecocentral scotoma (*n* = 8), nasal step (*n* = 2), global depression (*n* = 1), peripheral constriction (*n* = 1), arcuate scotoma (*n* = 1), and temporal wedge (*n* = 1). The results of OCT was not reported in any of the patient.

Eighty-eight percent of patients (15/17) tested positive for ANCA, including MPO-ANCA (*n* = 7), PR3-ANCA (*n* = 6), cANCA (*n* = 5), or pANCA (*n* = 1). Elevated CRP (*n* = 6) or ESR (*n* = 5) was found in 56% (9/16) of patients. CSF analysis showed abnormal results in most cases (6/7, 86%), including albuminocytologic dissociation (*n* = 3), increased immunoglobulin G index (*n* = 3), pleocytosis (*n* = 2), and a positive oligoclonal band (*n* = 1). Chest X-ray and CT were abnormal in eight patients (8/11, 73%), showing pulmonary nodules (*n* = 7), infiltrates (*n* = 1), or peribronchial thickening (*n* = 1). Urinalysis was abnormal in three patients (3/5, 60%), including gross/microscopic hematuria (*n* = 3) or proteinuria (*n* = 1).

MRI was abnormal in most patients (13/16, 81%, excluding one without detailed description) and included enhancement of the optic nerve sheath (*n* = 10) and optic nerve (*n* = 3). Abnormal T2-weighted signal intensity was also found in the optic nerve in three patients (3/16, 19%). Notably, pachymeningeal enhancement (*n* = 9) or thickening (*n* = 4) was found in 10 patients (10/16, 63%).

The patients were treated with intravenous or oral steroids (*n* = 13), cyclophosphamide (*n* = 9), methotrexate (*n* = 2), rituximab (*n* = 5), mycophenolate mofetil (*n* = 1), azathioprine (*n* = 1), or plasmapheresis (*n* = 1). The final visual outcome was often poor, with significant visual recovery maintained in only eight eyes (8/23, 35%). Recurrence was observed in 10 patients (10/17, 59%) from one to four times, either in the affected eye (*n* = 9) or fellow eye (*n* = 4).

## 4 Discussion

Our findings can be summarized as follows: (1) we report a patient and further identified 17 patients whose optic nerves were affected by GPA in isolation, without other ocular or adnexal involvement. (2) Eighty-two percent of the patients showed unilateral optic neuropathy initially, although sequential involvement of the fellow eye or recurrence was frequently observed. (3) Although remarkable visual improvement was documented in one-third, the final visual outcome was often not favorable, with visual impairment (only to count fingers) in 41% of the eyes affected. (4) ANCA positivity and optic nerve sheath or pachymeningeal enhancement on MRI aided in the discrimination of optic neuropathy associated with GPA from demyelinating optic neuritis. (5) Identifying other constitutional symptoms and signs of systemic involvement helped guide the diagnosis of optic neuropathy associated with GPA.

### 4.1 Optic nerve involvement in GPA

Ocular involvement is observed in nearly 40% of patients with GPA and is mostly associated with anterior segment inflammation (3). Scleritis and episcleritis are the most common manifestations, whereas optic neuropathy is reported in only 3% of patients (3). Its pathogenesis is usually explained by extension of granulomatous inflammation from the sinus or orbit (2, 3, 5). Therefore, when it occurs, it is mostly accompanied by strabismus, and the optic nerve is damaged due to compression by granulomatous inflammation (2, 5). Notably, ocular involvement is the first presentation of GPA in 14% of patients (3). Our findings further suggest that optic neuropathy can be an isolated ocular manifestation, without accompanying signs of other ocular or ocular adnexal involvement (Table 1).

### 4.2 Mechanism of optic nerve damage in GPA

The mechanism of isolated optic nerve damage is unclear in GPA. Although granulomatous inflammation is not evident, the optic nerve can be damaged by compression due to pachymeningitis (7). Alternatively, it can be ascribed to inflammation spreading from the adjacent sinuses to involve the optic nerves in the orbital apex, optic canal, and intracranial segment (8). Ischemic optic neuropathy secondary to small vessel vascilitis has been proposed a possible mechanism based on temporal artery biopsy findings of leukocytic infiltration, fibrinoid necrosis and occlusion of the small periadvential vessel (vasa vasorum) in one of the reports (9). Focal vasculitis may cause ischemia and infarction of the optic nerve and retina to resulting in pallid disc edema, as in our patient (2, 9, 10). The findings of optic nerve sheath and orbital enhancement on the MRI brain and orbits in our patient supports ischemia from posterior ciliary arteries as a possible mechanism for optic neuropathy. The rapidity and severity of the visual loss, the disc appearance (i.e., pallid disc edema), marginal response to steroids, and poor visual outcome also support ischemic optic neuropathy in our patient.

Our case presentation and literature review of similar cases suggest an interplay between inflammation and ischemia resulting in optic nerve damage in GPA. Optic nerve sheath and orbital inflammation can result in ischemic infarction of optic nerve, which can worsen inflammation from the release of proinflammatory mediators from the ischemic endothelium and activation of intravascular leukocytes (11–14).

Optic perineuritis seen as enhancement of the optic nerve sheath on MRI orbits are seen in a variety of infectious and inflammatory conditions such as, syphilis, sarcoidosis, giant cell arteritis, and GPA (15, 16). Although distinct from demyelinating optic neuritis, it is also found in demyelinating optic neuritis (especially myelin oligodendrocyte glycoprotein optic neuritis) (17–19). Patients with perineuritis show a dramatic response to steroid treatment and are more likely to experience relapse during tapering the dose or following discontinuation of treatment than patients with optic neuritis (15). Collectively, our results suggest that, even when other ocular or ocular adnexal involvement is not evident, clinicians should be wary when encountering patients with acute visual impairment with an atypical age of onset in the context of other system involvement, ANCA positivity and MRI findings associated with optic nerve sheath enhancement.

### 4.3 Differentiation of optic neuropathy associated GPA and typical optic neuritis

In most patients, differentiation of optic neuropathy associated with GPA and “typical” optic neuritis can be challenging at initial presentation (20, 21). Compared with those with a demyelinating etiology, visual acuity can be profoundly impaired (only to count fingers) (20). Alternatively, visual acuity can be preserved in case of the inflammation being confined to the optic nerve sheath. Similar to demyelinating optic neuropathy, disc changes are occasionally observed in GPA optic neuropathy (up to 24%) as inflammation affects the variable portion along the length of the intra-orbital to intracranial portion of the optic nerve (i.e., retrobulbar; Table 1) (20).

In this context, differentiation is difficult when relying solely on neuro-ophthalmological manifestations. Patients with GPA optic neuropathy usually do not fall the typical age range for demyelinating optic neuritis (22–24). They may also have additional systemic symptoms such as fever, weight loss and generalized weakness. MRI orbits may show optic nerve sheath enhancement in addition to optic nerve enhancement. Serological inflammatory markers of positive ANCA (88%), elevated ESR/CRP (56%) may also aid in differentiation. Notably, inflammation involving other organs (up to 94%, mostly involving the lungs and sinuses) can serve as red flags for systemic vasculitic inflammation. Given that abnormal findings are anticipated in 60–83% of patients, chest X-ray/CT and urinalysis should be obtained in cases of optic neuropathy with atypical presentation.

### 4.4 Caveats and limitations of our study and suggestions for future research

Our study has some limitations. First, the number of patients with isolated optic neuropathy in the GPA group was relatively small. This may lead to failure to assume a pathomechanism and limit the generalization of our findings. However, defining these patients has clinical implications because the diagnosis is based mostly on systemic signs and symptoms, which can be easily overlooked by neuro-ophthalmologists (25, 26). Second, because neuro-ophthalmological studies were not systematically conducted, the neuro-ophthalmologic findings were rather heterogeneous. It remains to be clarified which neuro-ophthalmologic sign, if any, can provide distinctive differential features in GPA optic neuropathy as opposed to demyelinating ON. Third, the literature review included studies based on the authors’ own diagnoses. We could not decide whether each patient fulfils the ACR/EULAR classification, since many of the findings were omitted or not specified in prior works. Our study emphasizes the importance of early detection of GPA-associated optic neuropathy, which can have a grave prognosis. We hope this case report and literature review will serve as a springboard for future studies with refined, structured evaluation and diagnosis.

Our findings indicate that optic neuropathy can present in the context of systemic inflammation of GPA without any other signs of ocular or orbital involvement. Identifying clinical, imaging, and laboratory signs of systemic inflammation can be important in cases of visual impairment with atypical presentation.

## Data Availability

The datasets presented in this article are not readily available because of ethical and privacy restrictions. Requests to access the datasets should be directed to the corresponding author.
